# Exploring sunflower responses to Sclerotinia head rot at early stages of infection using RNA-seq analysis

**DOI:** 10.1038/s41598-020-70315-4

**Published:** 2020-08-07

**Authors:** Mónica I. Fass, Máximo Rivarola, Guillermo F. Ehrenbolger, Carla A. Maringolo, Juan F. Montecchia, Facundo Quiroz, Francisco García-García, Joaquín Dopazo Blázquez, H. Esteban Hopp, Ruth A. Heinz, Norma B. Paniego, Verónica V. Lia

**Affiliations:** 1 Instituto de Agrobiotecnología y Biología Molecular (IABIMO), Instituto Nacional de Tecnología Agropecuaria (INTA), Consejo Nacional de Investigaciones Científicas y Técnicas (CONICET), Hurlingham B1686IGC, Buenos Aires, Argentina; 2 Instituto Nacional de Tecnología Agropecuaria (INTA). Estación Experimental Agropecuaria Balcarce, Balcarce, Argentina; 3 Bioinfomatics and Biostatistics Unit, Principe Felipe Research Center, Valencia, Spain; 4 Clinical Bioinformatics Area, Fundación Progreso y Salud (FPS), CDCA, Hospital Virgen del Rocio, 41013 Sevilla, Spain; 5 INB-ELIXIR-Es, FPS, Hospital Virgen del Rocío, 42013 Sevilla, Spain; 6 Departamento de Fisiología, Biología Molecular y Celular (FBMC), Facultad de Ciencias Exactas y Naturales (FCEyN), Universidad de Buenos Aires (UBA), 1428 Ciudad Universitaria, Buenos Aires, Argentina

**Keywords:** Genetics, Plant sciences

## Abstract

Sclerotinia head rot (SHR), caused by the necrotrophic fungus *Sclerotinia sclerotiorum*, is one of the most devastating sunflower crop diseases. Despite its worldwide occurrence, the genetic determinants of plant resistance are still largely unknown. Here, we investigated the Sclerotinia-sunflower pathosystem by analysing temporal changes in gene expression in one susceptible and two tolerant inbred lines (IL) inoculated with the pathogen under field conditions. Differential expression analysis showed little overlapping among ILs, suggesting genotype-specific control of cell defense responses possibly related to differences in disease resistance strategies. Functional enrichment assessments yielded a similar pattern. However, all three ILs altered the expression of genes involved in the cellular redox state and cell wall remodeling, in agreement with current knowledge about the initiation of plant immune responses. Remarkably, the over-representation of long non-coding RNAs (lncRNA) was another common feature among ILs. Our findings highlight the diversity of transcriptional responses to SHR within sunflower breeding lines and provide evidence of lncRNAs playing a significant role at early stages of defense.

## Introduction

Sunflower is one of the most important crops for the production of high-quality oil and seeds consumed by both humans and livestock. In recent years, sunflower production showed a steady increase driven by a boost in sunflower oil consumption (FAO, 2017). However, the projected expansion of the sunflower oil market requires appropriate agronomic management and improved genetic resources to cope with abiotic and biotic stresses. Among the latter, special attention should be paid to fungal diseases, as they have the greatest impact on yield and seed quality^1^.

The necrotrophic fungus *Sclerotinia sclerotiorum* is the causal agent of Sclerotinia head (SHR) and stalk (SSR) rots in sunflower. In particular, SHR is a recurrent disease in sunflower-growing areas worldwide. It affects oil quality and, under favourable conditions, may lead to total production loss^1,2^. Chemical fungicides proved to be ineffective and breeding of resistant genotypes has emerged as the most promising control strategy^3^. So far, there is no evidence of any major gene controlling the resistance to SHR in sunflower. Instead, inbred lines (ILs) show a broad range of responses in accordance with quantitative disease resistance (QDR) patterns depending on the genotype^4–8^. During the last 20 years, QTL mapping techniques have been used to unravel the complexity of the defense response to both SHR and SSR in sunflower. Biparental mapping has led to the discovery of several main effect loci and epistatic interactions^9–11^, whereas association mapping has served to identify candidate genes responsible for *S. sclerotiorum* resistance^12–14^. Notwithstanding this, little is known about the genetic architecture of quantitative resistance and the functional components of the defense response.

The fungus penetrates the host cuticle through mechanical means and by secreting an arsenal of cell wall-degrading enzymes, small proteins and secondary metabolite toxins, with oxalic acid being the major virulence factor. Diseased plants develop water-soaked lesions, tissue necrosis and finally bear sclerotia^15,16^, and in response to damage they can activate an array of perception mechanisms and signal transduction pathways that trigger QDR^17^. The polyphagous nature of *S. sclerotiorum*, which has been reported to infect over 400 plant species^18^, has allowed the identification of pathogen-responsive genes in a variety of model and economically important species. In line with current knowledge of the molecular mechanisms underlying plant immune response, transcriptomic studies in *Brassica napus*, *Arabidopsis thaliana* and *Glycine max* have shown that defense against *S. sclerotiorum* involves members of the WRKY transcription factor family, pathogenesis related (PR) proteins, as well as genes related to signal transduction, cellular redox state, cell wall composition and hormone signaling pathways^19–24^. However, there are no reports on the transcriptional response of sunflower to SHR.

Although SHR consistently causes water-soaked lesions and necrosis, the histopathology and time from infection to symptom onset differ among species^16^. In particular, sunflowers show no visible SHR symptoms for at least ten days after inoculation^6^, but microscopic lesions have been detected after a 24-h incubation period in susceptible and tolerant varieties, with the latter showing a slower progression of the disease^25^.

Transcriptional profiling at the site of infection provides an efficient means to reflect the natural epidemiologic cycle and constitutes an appealing alternative to discriminate among responses of genotypes with contrasting behaviour against the disease. RNA-sequencing (RNA-seq) has proven to be a powerful method to detect, map and quantify transcripts in several plant-pathogen interactions, regardless of the tissue under analysis or previous genomic knowledge^26^. A few studies conducted in cultivated sunflower have exploited the advantages and sensitivity of this methodology to identify differentially-expressed genes under biotic or abiotic stress conditions^27,28^, and none of them addressed the *S. sclerotiorum*-sunflower interaction. Indeed, only two low-throughput transcriptomic studies have focused on this pathosystem^29,30^. In addition, most transcriptomic analyses of *Sclerotinia* infection were made with data obtained from symptomatic plants^20–22^. However, transcriptional changes taking place during the asymptomatic period can provide insights into initial defense mechanisms and into the diversity of responses to the disease in different crops.

The aim of this study was to investigate the transcriptional response of sunflower during early stages of SHR. To this end, control and inoculated capitula of one susceptible and two tolerant ILs were subjected to RNA-seq at 0, 4 and 8 days post-inoculation (dpi) to analyze their expression patterns and gain a comprehensive view of the mechanisms involved in the defense against *S. sclerotiorum* infection.

## Results

### Disease assessment

ILs HA89, HA853 and RK416 were grown under field conditions during season 2010–11 at the experimental station INTA Balcarce (Buenos Aires, Argentina). After sampling for the RNA-seq experiments, additional plants of the different ILs were maintained in the field to evaluate disease evolution and confirm the efficacy of inoculation. Commercial hybrids ACA861 and Dekalb 3820 were used as susceptible and tolerant controls, respectively. The distinct symptoms of SHR were observed in all cases. The three ILs and the hybrids showed an increase in infection levels over time, as evidenced by both disease incidence (DI) and disease severity (DS) scores. DI values exhibited a fast increment in HA89 and ACA 861 and a slower escalation in HA853 and Dekalb 3820, whereas RK416 showed an intermediate behaviour. DS estimates revealed a rapid spread of the fungus in ACA 861, a slower advance in HA89, while RK416, HA853 and Dekalb 3820 showed a delayed expression of symptoms and did not reach the DS values of the first two cultivars (Supplementary Fig. S1).

### Overview of RNA-seq analysis

**Figure 1:**
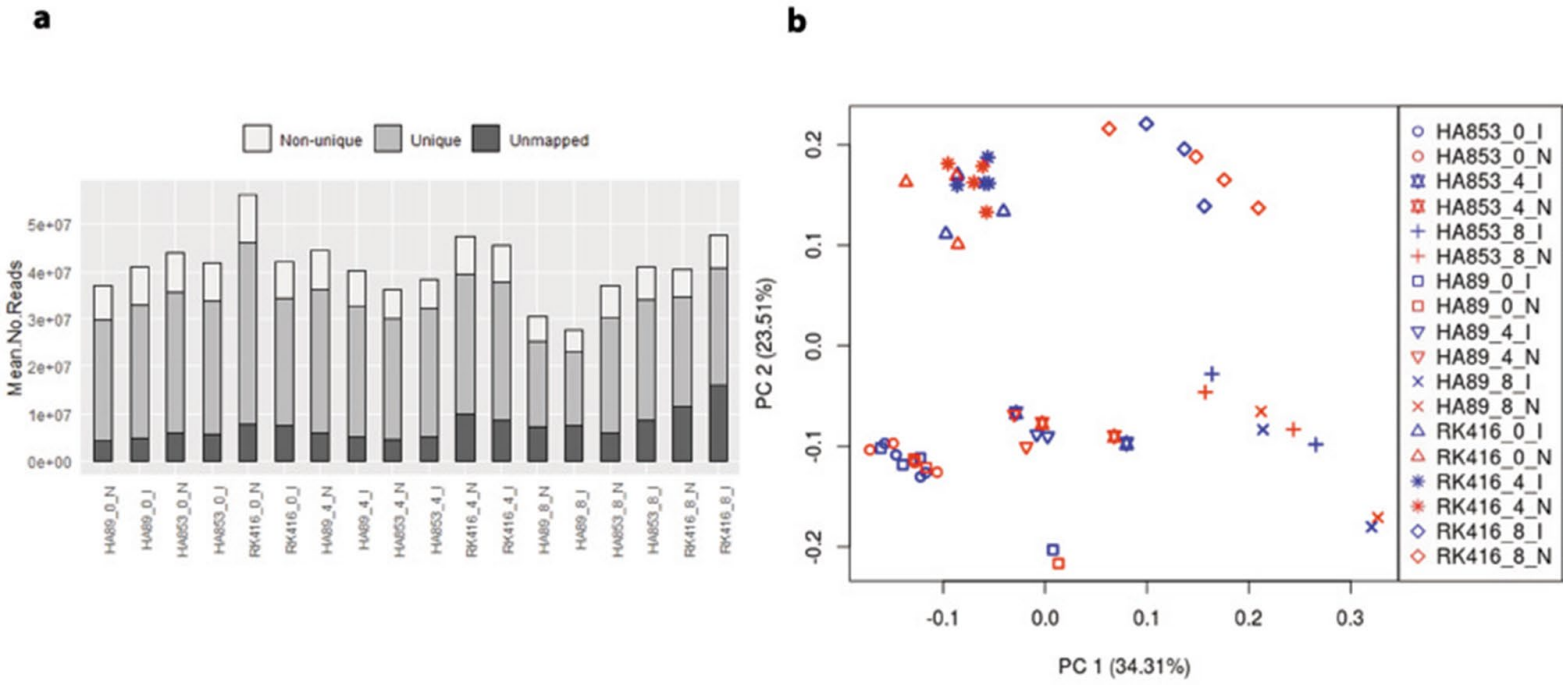
Overview of RNA-seq data. **(a)** Mean of Unique, Non-unique and Unmapped reads for each treatment and IL-time point combination. **(b)** Principal component analysis of 53 samples based on the expression levels of 31,673 transcripts.

Between 14.1 and 88.04 million reads per sample were mapped to the HanXRQ v1 sunflower genome (mean 42.18 ± 12.62 million reads). An average of 64.18 ± 5.91% and 17.62 ± 1.84% of reads were mapped uniquely and non-uniquely to the reference, respectively (Fig. 1a), whereas 18.19 ± 7.33% of the total reads could not be mapped and were excluded from the analysis (Supplementary Table S1).

Transcript expression levels were established at 0, 4 and 8 dpi in inoculated (I) and control (N) capitula of sunflower ILs HA89, HA853 and RK416 (Supplementary Table S2). Transcripts with more than 1 CPM in half of the samples were considered for further analysis, encompassing 31,673 of the 58,050 inferred genes annotated in the HanXRQ v1 sunflower genome^31^. Of these, 29,329 correspond to protein-coding genes (mRNAs) and 2,344 correspond to non-coding RNAs (ncRNAs).

Overall similarities between ILs and time point conditions were explored using PCA. Three samples from RK416 (4_18_RK416_0_I, 1_5_RK416_0_N and 2_11_RK416_8_I) were identified as outliers and thus removed from the analysis. In the final PCA, the first two principal components accounted for 57.82% of the total variation and grouped samples mainly by IL, separating RK416 samples from those of HA89 and HA853 (Fig. 1b). Within lines, samples were clustered according to the plant developmental stage (0, 4 and 8 dpi), irrespective of the inoculation condition, showing the greatest differences between 8 dpi and the previous time points. These results reveal large transcriptomic differences between RK416 and the other two ILs and imply an increasing time effect, consistent with the progression of disease symptoms.

### Analysis of differentially expressed genes

The expression levels of the 31,673 transcripts were estimated for each inoculation treatment and IL-time point combination. To validate these results, we assessed the correlation between RNA-seq transcription ratios (I/N) and those obtained in qPCR assays at 4 and 8 dpi. Between six and twelve genes were compared for each IL and time point. Only three genes were available for comparison in HA853 at 4 dpi, and therefore it was excluded from the analysis. In general, expression results from both methods were highly correlated, with coefficients ranging from 0.90 to 0.98 (p < 0.05). The only exception was HA89 at 8 dpi, which showed a weak and non-significant association (Supplementary Fig. S2).

**Figure 2:**
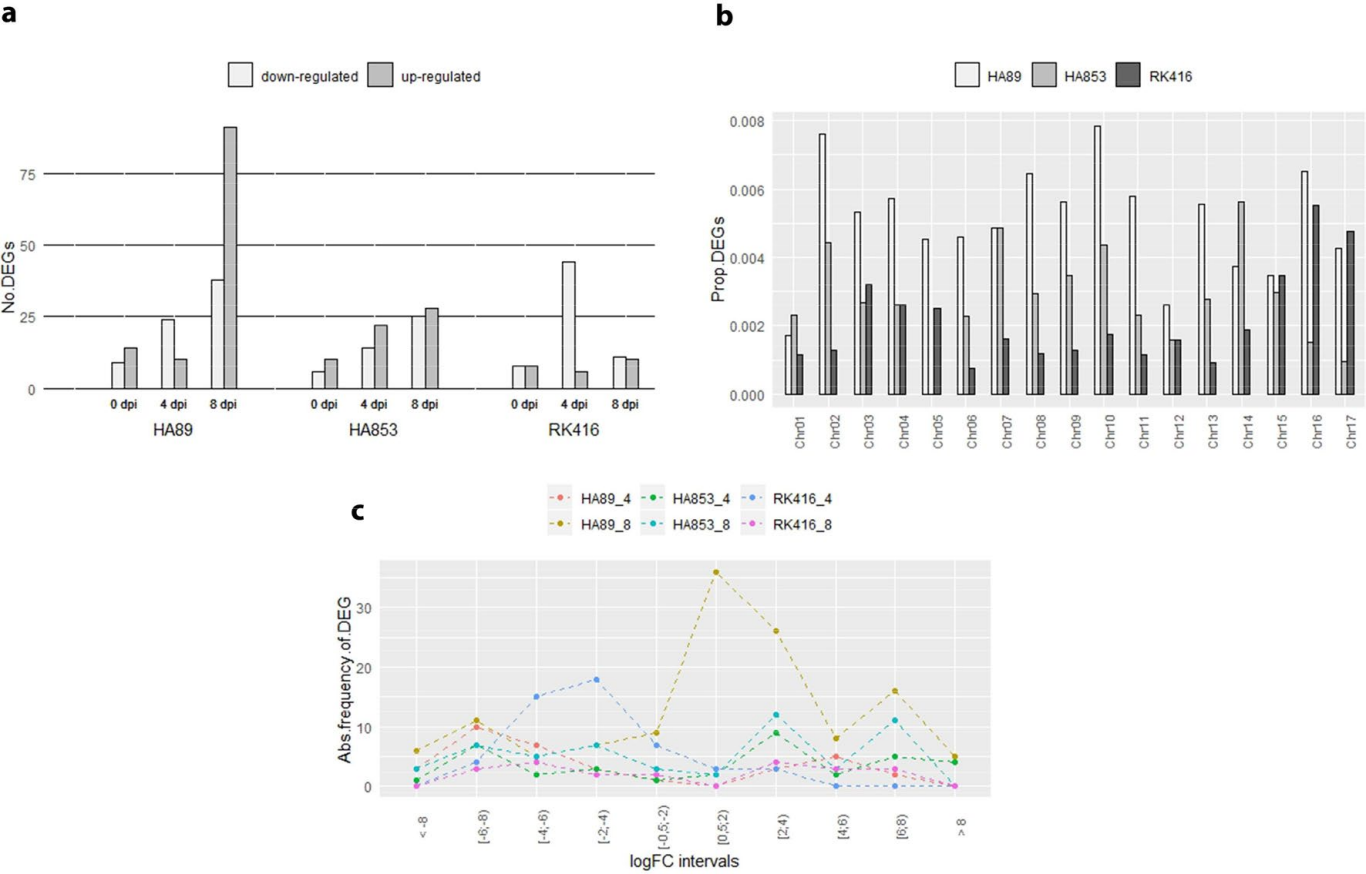
Analysis of DEGs. **(a)** Number of up- and down-regulated DEGs between I and N samples. **(b)** Proportion of DEGs relative to all expressed transcripts in each IL distributed across chromosomes. **(c)** Absolute Frequency of DEGs at different intervals of logFC values for each IL-time point combination (0 dpi not shown).

To minimise false positives due to multiple testing, only those genes with FDR < 0.05 were assessed and categorised as differentially expressed (DEGs) at each IL-time point combination. Differences at 0 dpi were also identified to quantify the level of noise generated from sources other than inoculation. A total of 378 DEGs were found, of which 186, 105 and 87 corresponded to HA89, HA853 and RK416, respectively (Supplementary Table S3). The following distribution of DEGs was obtained at 0, 4 and 8 dpi: 23, 34 and 129 for HA89, 16, 36 and 53 for HA853 and 16, 50 and 21 for RK416 (Fig. 2a). As expected, the number of DEGs was larger at 4 and 8 dpi than at 0 dpi. Most IL-time point combinations had similar numbers of up- and down-regulated DEGs. Notably, HA89 showed the largest proportion of up-regulated DEGs at 8 dpi and RK416 the largest proportion of down-regulated ones at 4 dpi.

The analysis of the distribution of DEGs across chromosomes indicated that all ILs presented DEGs in the 17 chromosomes, except for HA853 with no representation in chromosome 5. Chromosomes 2 and 10 had the highest number of DEGs, while chromosomes 1 and 12 had the lowest. Since HA89 encompassed the largest number of DEGs, these were the most abundant transcripts in 13 of the 17 chromosomes. Conversely, HA853 and RK416 showed a comparable amount of DEGs across the genome, except for chromosomes 16 and 17 where RK416 DEGs were more abundant (Fig. 2b).

In addition, to uncover distinct patterns of expression in the different IL-time point combinations, the distribution of the log_2_ fold change (logFC) for down- and up-regulated genes was analysed at 4 and 8 dpi (Fig. 2c). Overall values varied from |0.73| to |10.19|, irrespective of gene regulation. Inspection of Fig. 2c shows a large number of values within the lower intervals for HA89 at 8 dpi and more homogeneous patterns for the remaining IL-time point combinations.

The analysis of the DEG intersections among IL-time point combinations showed that there were only four DEGs in common between HA89 and HA853, albeit with opposite expression behaviour. Genes HanXRQChr04g0102281 and HanXRQChr13g0407331 were down-regulated in the susceptible line and up-regulated in the tolerant HA853 line. Conversely, genes HanXRQChr03g0066301 and HanXRQChr10g0282011 were down-regulated in HA853 and up-regulated in HA89. RK416 shared no DEGs with the other two lines. In addition, the intersection between time points within ILs included two DEGs for HA89, four for HA853 and none for RK416. A summary of these results is shown in Table 1.

**Table 1:** Functional description and logFC values of the DEGs shared by the different IL-time point combinations and/or time points within ILs.

Gene ID	Functional description	HA89_4	HA89_8	HA853_4	HA853_8
HanXRQChr04g0102281	Spliced ncRNA	–	− 7.45	–	2.97
HanXRQChr03g0066301	Transcription factor IBH1	–	2.35	–	− 1.69
HanXRQChr10g0282011	NA	–	7.38	–	− 6.79
HanXRQChr13g0407331	NA	–	− 1.42	7.55	–
HanXRQChr11g0350011	Retrovirus-related Pol poly from transposon TNT 1–94	− 6.91	7.89	–	–
HanXRQChr13g0418051	Mitochondrial import inner membrane translocase subunit TIM14-1	− 7.66	− 6.91	–	–
HanXRQChr10g0287891	NA	–	–	− 3.55	3.02
HanXRQChr14g0449141	Spliced ncRNA	–	–	6.37	− 6.46
HanXRQChr14g0435741	NA	–	–	− 7.00	− 6.27
HanXRQChr11g0331821	Alcohol dehydrogenase	–	–	9.32	− 9.64

Altogether, the ensemble of genes responding to the infection was IL-specific, suggesting the coordination of idiosyncratic reactions at early stages of the infection process.

To determine if DEGs were located close to previously reported QTLs^11,12^, and to reinforce the results obtained by both methodologies, we searched for DEGs in the vicinity of the molecular markers (MM) associated with SHR tolerance (Supplementary Table S4). Examination of these regions allowed the detection of five DEGs within the established 1 Mbp windows (Table 2). In detail, genes HanXRQChr03g0080031 and HanXRQChr03g0080021 were close to each other and within the range of MM HeAn_R_283.1, gene HanXRQChr10g0296411 was the closest to MM ORS437, gene HanXRQChr14g0449141 was in the vicinity of MM G34, while gene HanXRQChr14g0460321 was found near MM HaCOI1-1.

**Table 2:** DEGs located in the vicinity of QTLs associated with SHR resistance by biparental (BM) or association (AM) mapping.

DEG	IL-time point	Chr	Molecular marker (MM)	QTL source	Distance to MM (bp)	Functional description of DEG
HanXRQChr03g0080031	HA89_4	3	HeAn_R_283.1	BM	106,548	NA
HanXRQChr03g0080021	HA89_4	3	HeAn_R_283.1	BM	108,115	ncRNA
HanXRQChr10g0296411	HA853_8	10	ORS437	BM	7,475	DEAD-box ATP-dependent RNA helicase 40
HanXRQChr14g0449141	HA853_4-8	14	G34	AM	469,108	ncRNA
HanXRQChr14g0460321	HA89_8	14	HaCOI	AM	615,017	Hypoxia-responsive family

### Functional analysis

The functional annotation of DEGs was compiled from the HanXRQ v1 sunflower transcriptome^31^ (Supplementary Table S5). Gene Ontology (GO) Enrichment Analysis showed that no GO terms were significantly over-represented when strictly considering the DEG list. Despite the lack of enriched GO terms among DEGs in the different IL-time point combinations, at least one gene from each group was associated with defense processes. These include glutathione S-transferase DHAR3, chloroplastic-like (HanXRQChr13g0424161, HA89_4 dpi), probable leucine rich repeat (LRR) receptor-like serine threonine-kinase At1g07650 (HanXRQChr04g0123251, HA89_8 dpi), resistance RGC2, partial (HanXRQChr02g0057101, HA853_4 dpi), S-norcoclaurine synthase-like (HanXRQChr13g0397881, HA853_8 dpi), pathogenesis-related 1 (HanXRQChr04g0109991, RK416_4 dpi) and major allergen Pru ar 1-like (HanXRQChr03g0090261, RK416_8 dpi), among others^32–37^. Additionally, the MapMan ontology was used to organise the DEGs into main functional categories and relate them to possible processes occurring during the defense response to the infection (Supplementary Table S6 and Supplementary Fig. S3). Of the 34 specific functional categories (BINs) of the ontology, 23 were represented by at least one DEG. However, most DEGs fell within the “not assigned” category (BIN 35). The remaining DEGs were most frequently related to “protein” (BIN 29), “RNA” (BIN 27), “signalling” (BIN 30) and “transport” (BIN 34), in which the most common processes altered were protein degradation, regulation of transcription, as well as the transcriptional variation of receptor kinases. Relevant categories for the defense response included “cell wall” (BIN 10), “hormone metabolism” (BIN 17) and “stress” (BIN 20), which were represented in all ILs. Overall, our results revealed that although there was modulation of similar processes among ILs, they were mediated by different biological molecules.

To extend the exploration of enriched GO terms, a logistic regression was applied on the whole gene set, indexed according to logFC values and the adjusted p-value. This Gene Set Enrichment Analysis (GSEA) approach yielded a total of 1,128 over-represented GO terms (biological process (BP): 704; cellular component (CC): 227; molecular function (MF): 197) (Supplementary Table S7). RK416 presented the largest number of enriched GO terms, followed by HA853 and HA89. The latter two lines showed only a few enriched GO terms at 4 dpi (55 and 26, respectively), but these increased three to ten times at 8 dpi. In comparison, RK416 maintained a similar number of enriched GO terms at both time points.

There was little overlapping of enriched GO terms among IL-time point combinations. Only 15% of GO terms were shared by at least half of the combinations, and just a few were shared by five or six of them (i.e., photosynthesis, BP; photosynthesis, light reaction, BP; ribosome biogenesis, BP; cytosolic part, CC; ribosomal subunit, CC). After checking for redundancy, 699 unique GO terms were identified (BP: 455; CC: 114; MF: 130).

**Figure 3:**
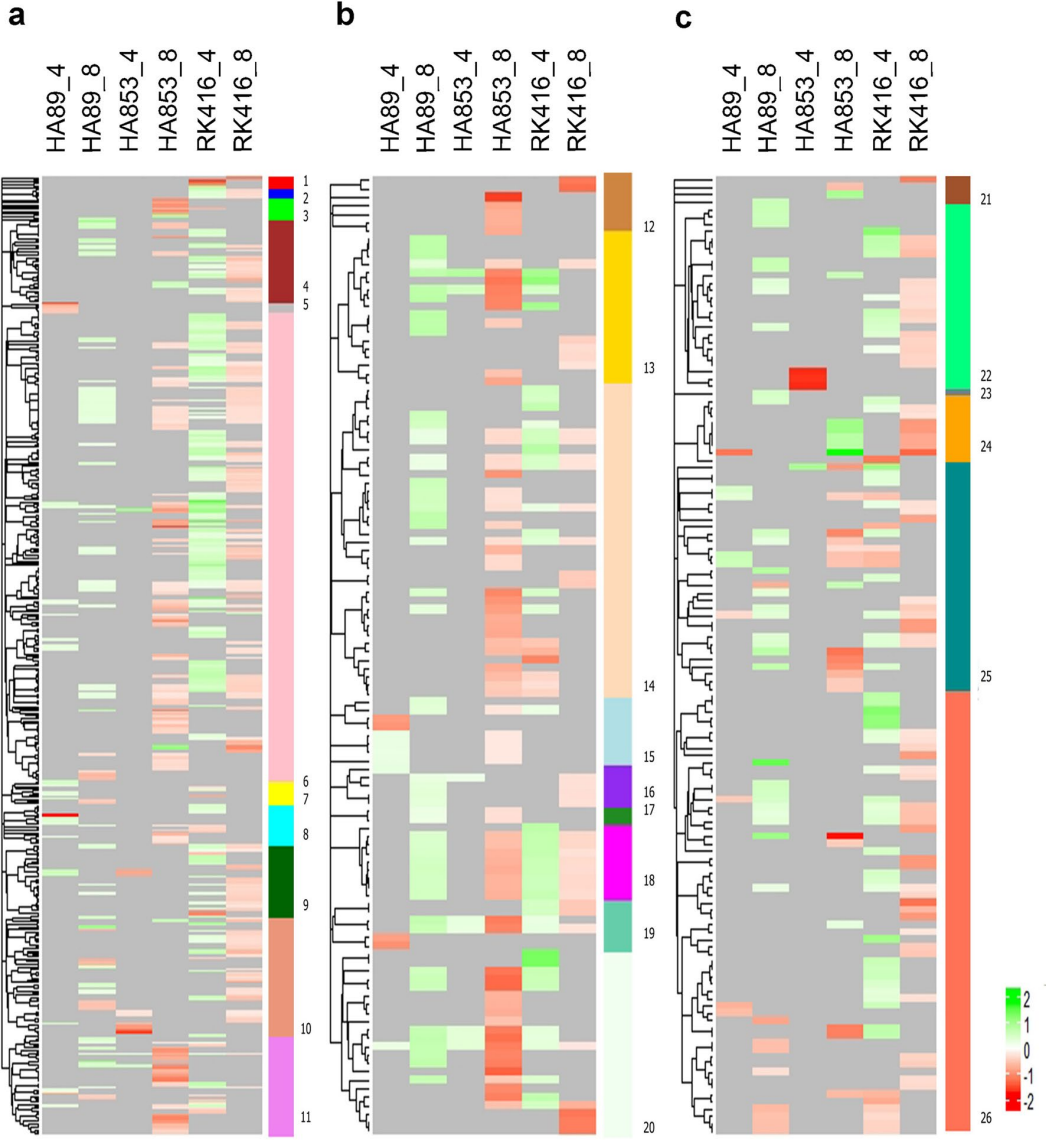
Heatmaps based on the LOR values of GO terms grouped by semantic similarities. Functional terms over-represented in up-regulated genes (green) are differentiated from those over-represented in down-regulated genes (red) upon inoculation. **(a)** BP ontology, **(b)** CC ontology and **(c)** MF ontology. Vertical coloured bars represent functional modules in which GO terms were categorised. 1: miscellaneous processes; 2: immune system process; 3: miscellaneous organisation; 4: transport and localisation; 5: reproductive development; 6: metabolic process; 7: developmental process; 8: cell integrity; 9: biological regulation; 10: response to stimuli; 11: cellular organisation and biogenesis; 12: miscellaneous CC ; 13: membrane and membrane-related complexes; 14: organelles; 15: extracellular region and cell junction; 16: Golgi and ER; 17: encapsulating structure; 18: thylakoid; 19: lytic organelles; 20: protein-containing complexes; 21: miscellaneous MF; 22: transporter activity; 23: antioxidant activity; 24: molecular function regulation; 25: binding; 26: catalytic activity.

Figure 3 shows the dendrogram derived from the GO term similarity matrix obtained with GoSemSim, with the corresponding LOR plotted as a heatmap for each IL-time point combination. The LORs of the different GO terms were assigned by Multidimensional Gene Set Analysis^38^ based on the expression levels of the genes included within each category. It is noteworthy that the functional categories over-represented in HA89 at 8 dpi and RK416 at 4 dpi were mainly derived from up-regulated genes, while HA853 and RK416 at 8 dpi showed the opposite pattern.

The classification of GO terms according to their GoSemSim similarities allowed the delimitation of functional modules (Fig. 3, Supplementary Table S8). In this way, apparently unconnected GO terms were grouped into more general processes, thus becoming comparable between the different IL-time point combinations. In regard to BP, “metabolic processes” was the main functional module, i.e. the one including the largest number of related GO terms, and, to a lesser extent, “response to stimuli” and “cellular organisation and biogenesis” were also frequently represented. In the CC category, the main functional module was “organelles”, closely followed by “protein-containing complexes” and “membrane and membrane-related complexes”. As for MF, the main functional module was “catalytic activity”. Secondary modules included “binding” and “transporter activity”. In general, functional modules consisted of GO terms from all the IL-time point combinations, indicating a modulation of similar general processes but with different actors being involved in each condition.

A gene set enrichment analysis based on the Kyoto Encyclopedia of Genes and Genomes (KEGG) database revealed two enzyme KEGG identifiers (EC) over-represented in HA89 at 8 dpi, both belonging to Class “Oxidoreductases”. One EC from the class “Hydrolases” was identified in HA853 at 8 dpi, whereas eight EC were identified in RK416 at 4 dpi, half of them being “Transferases” and the other half “Hydrolases”. Finally, one EC classified within “Oxidoreductases” was identified in RK416 at 8 dpi (Supplementary Table S9). Although all of them can be related to defense processes, the enzymatic reactions identified in this analysis were not completely concordant among ILs.

### Study of differentially expressed ncRNAs in response to *S. sclerotiorum* infection

**Figure 4:**
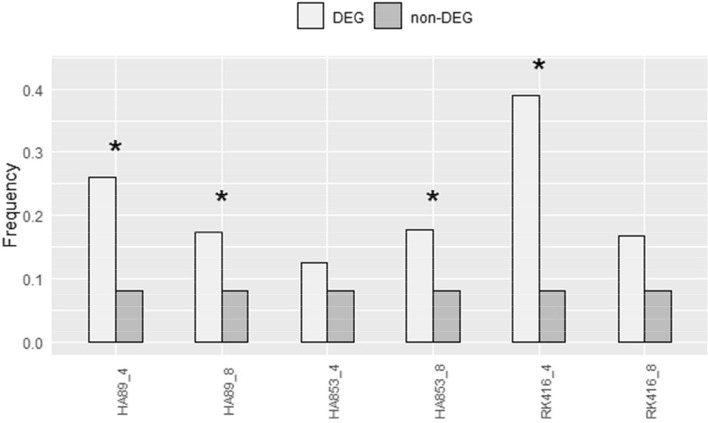
Analysis of ncRNA enrichment for the different treatments; *: p < 0.05.

Taking into account that ncRNA genes are not part of GO terms, but that they were frequently represented in the IL-time point combinations, their enrichment in the different groups was examined through Irwin-Fisher tests (Supplementary Table S10). The proportion of differentially expressed ncRNAs compared to that of the remaining ncRNAs was significantly higher in HA89 at both time points, in HA853 at 8 dpi and in RK416 at 4 dpi (p-value < 0.05) (Fig. 4). According to the HanXRQ v1 annotation, all ncRNAs corresponded to the splice ncRNA category. They were considered as long ncRNAs (lncRNAs) based on the length of the identified transcripts (> 200 bp).

Because lncRNAs are involved in transcriptional and post-transcriptional regulation of protein-coding RNAs, we used the LncTar algorithm to predict putative mRNA targets among DEGs. This tool computes the normalised binding free energy (ndG) between molecules, thereby estimating a putative interaction. Here, differentially expressed protein-coding genes and lncRNAs of the same IL at 4 and 8 dpi were used as input to predict their binding capability. We identified one possible mRNA-lncRNA interaction for RK416, nine for HA89 and none for HA853 (Table 3). Of these, six pairs were differentially expressed at different time points and four at 8 dpi. Additionally, only four pairs showed the same expression behaviour.

**Table 3:** Putative lncRNA-mRNA interactions within DEGs.

Inbred line	lncRNA			mRNA/target			ndG	Functional description of mRNA
	Gene ID	dpi	Fold-change	Gene ID	dpi	Fold-change		
RK416	HanXRQChr01g0005231	8	− 2.68	HanXRQChr14g0427881	4	− 4.46	− 0.4193	NA
HA89	HanXRQChr08g0221721	4	2.44	HanXRQChr02g0038901	8	− 4.74	− 0.7460	NA
HA89	HanXRQChr08g0226611	4	− 8.97	HanXRQChr08g0235441	8	− 6.13	− 0.2542	NA
HA89	HanXRQChr08g0229491	4	5.07	HanXRQChr02g0038901	8	− 4.74	− 0.5924	NA
HA89	HanXRQChr16g0506901	4	4.72	HanXRQChr17g0559391	8	− 6.99	− 0.2190	Quinone oxidoreductase 2 homolog
HA89	HanXRQChr02g0033591	8	− 2.00	HanXRQChr02g0046121	4	− 1.58	− 0.6558	NA
HA89	HanXRQChr08g0226611	8	− 8.97	HanXRQChr10g0314321	8	3.06	− 0.1667	E3 UFM1- ligase 1 homolog
HA89	HanXRQChr13g0406311	8	3.64	HanXRQChr02g0041971	8	− 7.42	− 0.1620	Mitogen-activated kinase 16, partial
HA89	HanXRQChr13g0406311	8	3.64	HanXRQChr02g0046121	8	− 1.58	− 0.2095	NA
HA89	HanXRQChr13g0406311	8	3.64	HanXRQChr10g0308951	8	2.28	− 0.1504	PREDICTED: uncharacterised protein LOC103323638

*ndG* normalised free energy, *NA* not available.

Although such interactions need further experimental confirmation, these results provide a first approach to assess the involvement of lncRNAs in sunflower defense processes.

## Discussion

The identification of the genes underlying quantitatively inherited traits is one of the most challenging tasks for molecular breeders. Nonetheless, gene pyramiding strategies are considered the most effective way to achieve durable resistance^39–41^. Large-scale transcriptional studies are a fruitful source of novel candidate genes, as well as a means of validating previous results obtained by other approaches. By conducting a genome-wide transcriptomic analysis of one susceptible and two tolerant sunflower ILs to SHR, we were able to uncover new molecular determinants of defense under conditions that mimic the natural infection process.

In this study, we established the expression profiles of more than half of the annotated genes in the sunflower genome across ILs, inoculation treatments and time points. However, the number of DEGs identified here was relatively low compared to those generally reported in RNA-seq analyses conducted for other pathosystems (e.g. Gao et al., Kamber et al., Song et al., Wu et al.^42–45^). At early stages, SHR is restricted to a small portion of the inflorescence. Therefore, RNA extraction from the whole set of florets in each capitulum may have diluted the biological signal, limiting our ability to detect the full range of transcriptional changes triggered by the pathogen. Although our approach may have prevented the detection of many of the small expression changes expected at the onset of infection, when no symptoms are visible^46,47^, it is likely to have detected more reliable and stronger expression variations. Indeed, several DEGs were readily identified at 4 dpi in all ILs, indicating a rapid activation and amplification of plant defense responses.

A comparison of gene activity elicited upon infection revealed limited commonalities among the three ILs and between time points within ILs. HA89 and HA853 were the only ILs that shared DEGs and the fact that these had opposite expression suggests a converging pathway leading to different responses. Of the four genes, one is described as a transcription factor (IBH1) involved in the modulation between growth and immunity in *A. thaliana*^48^, another one is inferred to be a ncRNA, while the remaining two have no known function. The singular behaviour of these genes may be indicative of their relevance in the defense pathway, making them promising candidates for improving resistance^24,49^. On the other hand, although HA853 and RK416 have been characterized as tolerant to SHR^6^, they had no DEGs in common. The comparison of time points within ILs revealed two and six DEGs at 4 and at 8 dpi in HA89 and in HA853, respectively. The sustained differential expression of these genes suggests a modulation over time, in contrast to the time-specific response observed in most DEGs. Against expectations, more DEGs were detected at 4 dpi than at 8 dpi in RK416, which may indicate an earlier activation of defense mechanisms. Altogether, tolerant lines appear to deploy different responses to fungal attack. This differential pattern of defense is concordant with the diverse origins of the ILs used in this study and with the dynamics of the sunflower domestication process, which included a larger number of genes with a small phenotypic effect than in other domesticated plant species^50^. Although the three ILs examined here are maintainer lines, HA89 and HA853 originated in USA, whereas RK416 originated in Argentina. HA89 is derived from the Russian domestic oilseed variety “Vniimk 8931”^51^, and HA853 from the “1975 High Yield Composite”, which in turn was generated from 11 high-oil Russian cultivars^52^. RK416 was developed by crossing the Composite Ruso, derived from Smena, Arnavirsk, Peredovik and Vniimk open pollinated populations, with the variety Klein, an Argentinian population based on Russian varieties, which were brought by Jewish immigrants as confectionery seeds (Julio Gonzalez, pers. comm.).

It is known that *Helianthus* species possess a rich spectrum of non-redundant defense mechanisms, many of which were introduced into cultivated sunflower from their wild relatives^53,54^. QTL mapping has served to confirm the diversity of SHR tolerance sources within breeding germplasm^4,11,13,55^, whereas combining transcriptomic and QTL mapping data has recently emerged as a powerful tool to identify candidate genes^24,56–58^. Our transcriptomic study showed that DEGs were homogeneously distributed throughout chromosomes, as expected for a complex quantitative trait. Interestingly, the absence of a cluster of DEGs also suggests a staggered introgression of defense responses, instead of large wild-relative introgression blocks derived from modern breeding. In addition, the results presented here allowed to refine the mapping resolution of the recent QTL analysis by identifying five DEGs that co-localised with QTL regions. Functional annotations of these DEGs include one DEAD-box ATP-dependent RNA helicase 40, one hypoxia-responsive family protein and two ncRNAs, one of which was differentially expressed at 4 and 8 dpi in HA853, and the other has no known function. DEAD-box helicases have been identified as key factors of abiotic stress responses in plants^59^, and hypoxia-responsive proteins have been related to group VII Ethylene Response Factors, which in turn comprises elements playing a central role in defense against necrotrophic pathogens^60–62^. These putative functions, together with their relationship to previously identified QTLs, highlight the potential of these genes as candidates to improve resistance.

In this work, different functional approaches were undertaken to explore the molecular responses to SHR. Regardless of the method employed, each IL-time point combination showed a unique array of over-represented GO terms and catalytic enzymes. This suggests a differential contribution of IL-specific features to tolerance and susceptibility, and the existence of alternative control strategies involved in cell defense response. Further grouping of GO terms into broad functional categories revealed that ILs shared common mechanisms (i.e. responses to stimuli, catalytic processes or altered transport activities).

Two types of resistance have been described in sunflower: resistance to penetration and to mycelial spread in tissues^63^. The type of resistance is likely modulated by the transcriptomic responses elicited upon infection, as they represent the first line of defense and determine the outcome. The functional annotations of the DEGs identified here, as well as their enriched GO terms, may be related to both resistance processes. In tems of QDR, many causal genes are responsible for the defense response and act beyond pathogen recognition^64^. The perception of the pathogen frequently involves LRR receptor-like kinases (RLKs), which propagate external signals through their kinase domain^32,65^. Signal transduction pathways are then mediated by kinase/phosphatase activity and the alteration of ion fluxes, such as Ca^2+^^66,67^. To counteract pathogen infection, these signaling networks regulate the activity of transcription factors, transcriptional machinery, enzymes and antimicrobial compounds, including pathogenesis-related (PR) proteins^68–71^. In accordance with this general mechanism, a putative LRR RLK was found in HA89, while candidates involved in signaling networks or transcription factors were detected in HA89 and HA853. In addition, putative PR1 and PR10 proteins were found down-regulated in RK416 at 4 and 8 dpi. As suggested by Cregeen et al. and Upadhyay et al.^72,73^, the down-regulation of PR proteins may be responsible for the delay in the appearance of symptoms observed in tolerant plants, by blocking fungal spread. Since expression of PR1 is related to a salicylic acid (SA) response, it can also be inferred that the SA-pathway is suppressed upon infection in the tolerant IL RK416^74^.

In agreement with current knowledge about the initiation of plant immune responses, all three ILs altered the expression of genes involved in the cellular redox state^75,76^. In addition, the studied ILs appear to reorganise their cell wall composition and use extracellular compounds during defense. We also found genes and GO terms directly associated with defense responses, particularly in RK416 and, to a lesser extent, in HA853 (e.g. a resistance gene candidate 2^34^; a S-norcoclaurine synthase-like gene^35^; or a Universal stress protein A^77^). Overall, the functional analysis reinforces the idea of genotype-specific activation of defense pathways. Based on the DEGs and the GO terms enriched in the different ILs, RK416 exhibits the widest arsenal of resources to resist fungal penetration, at least in the environment in which our experiment was performed.

Noteworthy, the most consistent signal in the defense response of sunflower to SHR seems to be related to ncRNAs. Two out of the five DEGs found in the vicinity of previously identified QTLs are ncRNAs, two out of ten DEGs shared by the different IL-time point combinations are ncRNAs, and the ncRNA category is significantly enriched in four out of the six IL-time point combinations. A survey of ncRNA studies has shown that genes encoding RNAs, rather than proteins, have both structural and regulatory functions^78,79^. In this study, we could only assess spliced ncRNAs over 200 nt (i.e. long ncRNAs) due to a bias towards the sequencing of polyadenylated RNAs. LncRNAs are poorly conserved and display diverse synthesis, processing and regulatory functions. In plants, lncRNAs can function as gene^80–82^, transcription^83–85^, or epigenetic regulators^86^. Moreover, they are known to participate in basal defense against stresses, including response to pathogenic fungi, where they act as precursors of sRNAs, microRNAs (miRNAs) and/or small interfering RNAs (siRNAs)^87–91^. Recently, it was found that *A. thaliana* cells secrete exosome-like extracellular vesicles to deliver sRNAs into *Botrytis cinerea*, a fungal necrotrophic pathogen affiliated to *S. sclerotiorum*. The sRNAs transferred by the host induce silencing of fungal genes essential for pathogenicity^92^.

Although ncRNAs appear to play a significant role as a first line of defense to SHR in sunflower, the specific mechanisms by which they operate are yet to be determined. Analysis of RNA–RNA interactions revealed possible mRNA targets within DEGs, but RNA–DNA or RNA–protein interactions cannot be ruled out. In particular, the genes HanXRQChr03g0080021 and HanXRQChr03g0080031, coding a lncRNA and a mRNA, respectively, could represent an example of a RNA–DNA interaction, as their vicinity may facilitate a cis-acting regulatory function of transcription^79,93^.

The relationship between tolerance to SHR and the expression patterns observed in this study is still difficult to determine. In a recent multi-environment trial, RK416 and HA853 ranked among the top 20 of 137 ILs, with RK416 showing lower DI and DS scores than HA853^6^. These findings were only partially replicated in our field trial, where HA853 outperformed RK416, although both exhibited a similar behaviour to that of the tolerant control Dekalb 3820. In line with these observations, sunflower response to SHR is known to be greatly affected by environmental variables such as temperature, humidity, and rainfall^6^. In this context, our results suggest that the ensemble of DEGs identified here may only partially represent the full array of sunflower responses to SHR, with differences in environmental conditions triggering additional reactions, even for the same IL.

In sum, this research represents the first report on the transcriptional changes occurring in sunflower upon infection with *S. sclerotiorum*. Our results suggest genotype-specific defense responses, in line with the diversity of tolerance levels observed under field conditions^6^. The combined analysis of transcriptomics and QTL mapping reported here provides a promising approach to identify relevant genes and genomic regions associated with QDR. Finally, the involvement of ncRNAs adds an unexpected complexity to the discovery of the genetic determinants of SHR resistance.

## Methods

### Plant material and field trial

Three sunflower public ILs were used in this study: HA89, HA853 and RK416. Plants of each IL were grown under field conditions during season 2010–11 at the experimental station INTA Balcarce (Buenos Aires, Argentina). A randomised complete block design comprising three blocks with a furrow per IL was used. Furrows were 6 m long and spaced 75 cm apart. Commercial sunflower lines ACA861 and Dekalb3820 were included in each block as susceptible and tolerant controls, respectively.

Plants that reached the R5.2 flowering stage of the Schneiter and Miller^94^ scale on the same day were selected in pairs and capitula were ascospore- (2,500 ascospores/ml) (I) or mock- (water) (N) inoculated with 1 ml of inoculum using a portable hand sprayer^95^. Ascospores were obtained by inducing carpogenic germination of sclerotia, as described by Escande et al.^95^. Briefly, apothecia were produced from sclerotia collected at the end of the 2009/2010 field trials from naturally and experimentally infected plants at agricultural station INTA Balcarce. Mature apothecia were hold in Petri dishes, incubated for 4 h to favor ascospore release and finally stored at − 18 °C until use. Inoculated capitula were collected at 0 (i.e. immediately after spraying), 4 and 8 dpi, resulting in a total of 56 samples. Four biological replicates at 0 dpi and two biological replicates at 4 and 8 dpi were analysed for HA853 and HA89, whereas four biological replicates were used for RK416 at all time points (Supplementary Table S1). Time 0 after inoculation was included to assess the incidence of false positives in the statistical analysis.

Given that SHR symptoms appear at least after 10 dpi^6^, 14 to 21 plants per block and IL were maintained in the field to evaluate disease progression and confirm the effect of the inoculation protocol.

### Disease assessment

Disease progression was quantified by estimating the phenotypic variables DI, i.e., the number of plants infected over the number of plants inoculated in each furrow, and DS, i.e., the average proportion of capitulum rotted area of plants inoculated in each furrow. These variables have been described as suitable for appraising the resistance to fungal penetration in the first case, and the resistance to the spread of the fungus in the second case^96^.

For graphical visualization of the results, average DI and DS values per day post inoculation and IL were plotted using GraphPad Prism, v 5.01 for Windows, GraphPad Software, La Jolla California USA, https://www.graphpad.com (Supplementary Fig. S1).

### RNA isolation and sequencing

Disc florets and bracts were scraped off each capitulum and grinded in liquid nitrogen. Total RNA of 100 mg grinded material was extracted using the RNAqueous Kit with addition of the Plant RNA Isolation Aid (Ambion, Applied Biosystems, USA). Samples were treated with DNase I (Invitrogen, USA) for 20 min to remove remaining DNA. Sample purity, integrity and concentration were assessed using the RNA 6000 Nano Reagent kit in a 2100 Bioanalyzer (Agilent Technologies, Palo Alto, California, USA).

Sequencing libraries were prepared using an Illumina TruSeq RNA Sample Preparation Kit following the manufacturer’s instructions. Libraries were sequenced using the Illumina HiSeq 2000 system at the Centre Nacional d'Anàlisi Genòmica (CNAG: https://www.cnag.crg.eu/, Barcelona, Spain) as unstranded single-end reads of 50 bp in length. The original data set was deposited at the NCBI Sequence Read Archive (Submission ID: SUB5575431, BioProject ID: PRJNA561716).

### Analysis of RNA-seq data

Low-quality bases (average Q-score below 20) and adaptor sequences were trimmed and sequences with less than 36 bases long were removed with Trimmomatic^97^. The remaining reads were mapped to the *Helianthus annuus* XRQ (HanXRQ v1) genome-derived transcriptome (https://www.heliagene.org/HanXRQ-SUNRISE/)^31^ with Bowtie2, using the global sensitive parameter and allowing all multimapping reads to be mapped^98^. The EM algorithm of eXpress was used for transcriptome quantification^99^. Differential gene expression analysis was performed with the program edgeR^100^. In summary, transcripts with < 1 CPM in half of the samples were filtered out and then normalised using the trimmed mean of M-values (TMM) method. Read counts for each gene were fit to a negative binomial generalised log-linear model using the glmFit function, defined as:

$${\text{Y}}_{{{\text{ijkl}}}} = \, \mu \, + {\text{ G}}_{i} + {\text{ D}}_{j} + {\text{ T}}_{k} + {\text{ L}}_{l} + {\text{ GDT}}_{jik} + \, \varepsilon_{ijkl}$$

which includes the IL (G) i, the day post inoculation (D) j, the inoculation treatment (T) k and the sequencing lane (L) l as factors, and the interaction among G, D and T.

To identify DEGs, genewise likelihood ratio tests between I and N samples were conducted using the glmLRT method for each IL-time point combination. Transcripts were considered as differentially expressed between ascospore- and mock-inoculated treatments if the absolute value of logFC was ≥ 0.6 and FDR was < 0.05.

### Validation of RNA-seq analysis by qPCR

qPCR assays, which were previously performed to validate a microarray experiment conducted on the same samples as our RNA-seq analyses, were used to confirm the transcript levels estimated here.

Briefly, cDNA for qPCR was synthesised using Superscript III first strand synthesis system (Invitrogen, USA) and random hexamer primers according to the manufacturer's instructions. For amplification, a 25-µl reaction mix containing 200 nM of each primer, 1 µl of cDNA sample and FastStart Universal SYBR Green Master (Roche Applied Science, Germany) was run in a 96-well plate thermocycler (ABI Prism 7000 Sequence Detection System and software, PE Applied Biosystems, USA). Amplification efficiencies and Ct values were determined for each transcript using LinRegPCR^101^. The relative expression of the genes was determined using the “RT-PCR comparison of relative gene expressions analysis” included in the InfoStat software^102^. Actin was established as internal standard after comparison with Elongation Factor-1α and Tubulin using the program Bestkeeper^103^. Primer pairs used for the qPCR assays are listed in Supplementary Table S11.

### Co-localisation of DEGs to previously identified QTLs

To investigate the putative correspondence between previously reported QTLs and the DEGs identified here, 39 MM associated with SHR resistance^11,12^ were mapped to the HanXRQ v1 genome^31^ using primersearch v6.6.0.0 for in silico PCR^104^. DEGs falling within a window size of ± 1 Mbp around the MM were considered to co-localise with the QTLs (Supplementary Table S4).

### Functional profiling of genes

To assess the over-representation of GO categories, Single Enrichment analyses were performed on DEGs from each IL and time point using FatiGO^105^.

Mapman classification^106^ was used to interpret DEGs in the context of hierarchically organised cellular pathways and processes focused on plant metabolism^107,108^. To enable mapping, the genes predicted in the HanXRQ v1 sunflower genome annotation were re-annotated through the Mercator pipeline using *A. thaliana*’s functional annotation^109^, following Moschen et al.^110^.

In addition, a GO GSEA based on logistic regression was also applied to the whole gene set using the uvgsa function of the mdgsa R package^38^, with transcripts indexed by the logFC and the adjusted p-value. Enrichment was considered significant for p < 0.05, after Benjamini & Yekutieli^111^ correction for multiple testing. To evaluate the affiliations among the over-represented GO terms, semantic similarities were computed using the goSim function of the GOSemSim R package^112^ based on the genome-wide annotation for *Arabidopsis*^113^. Terms not identified in this database were given zero similarity relative to the other terms. GO terms were plotted on heatmaps according to their LOR values and grouped by semantic similarities using the complete-linkage hierarchical clustering method implemented in the R package ComplexHeatmaps.

Finally, to explore systems and chemical information collected in the KEGG database, a KEGG gene set analysis was also performed using the uvgsa function as described previously.

All analyses were performed based on the HanXRQ v1 gene annotation from Badouin et al.^31^.

### Analysis of differentially expressed non-coding RNAs

To include ncRNAs into the functional enrichment analysis, the number of differentially expressed ncRNAs of each IL-time point combination was compared to the total number of expressed ncRNAs by bilateral Irwin-Fisher tests using InfoStat^102^.

The LncTar software, version of November 10, 2015^114^ was used to predict the interaction between the ncRNAs and the mRNAs differentially expressed in each IL. Type 1 command line was used to perform all ncRNAs- vs. all mRNAs predictions. Following the developers’ recommendations, a ndG cutoff of − 0.15 was used to obtain high confidence predictions.

## Supplementary information

Supplementary Information 1.

Supplementary Information 2.

Supplementary Information 3.

Supplementary Information 4.

Supplementary Information 5.

Supplementary Information 6.

Supplementary Information 7.

Supplementary Information 8.

Supplementary Information 9.

Supplementary Information 10.

Supplementary Information 11.

Supplementary Information 12.

## Data Availability

All data generated or analysed during this study are included in this published article, its Supplementary Information files or deposited at the NCBI Sequence Read Archive (Submission ID: SUB5575431, BioProject ID: PRJNA561716).
