# Supplementary material for: Exploring sunflower responses to Sclerotinia head rot at early stages of infection using RNA-seq analysis
Source: Sci Rep. 2020 Aug 7;10:13347. doi: 10.1038/s41598-020-70315-4 (PMC7414910; doi:10.1038/s41598-020-70315-4)
Supplement: Supplementary file 1 — Supplementary Information 1. [file 41598_2020_70315_MOESM1_ESM.pdf]

Exploring sunflower responses to Sclerotinia Head Rot at early stages of infection using RNA-seq analysis

Mónica I. Fass [1, 2 †*], Máximo Rivarola [1, 2 †], Guillermo F. Ehrenbolger [1, 2], Carla A. Maringolo [3], Juan F. Montecchia [1, 2], Facundo Quiroz [3], Francisco García-García [4], Joaquín Dopazo Blázquez [5,6], Horacio E. Hopp [1,2], Ruth A. Heinz [1,2], Norma B. Paniego [1,2 γ]., Verónica V. Lia [1,2 γ]

[1]Instituto de Biotecnología, Centro de Investigaciones en Ciencias Agronómicas y Veterinarias, Instituto Nacional de Tecnología Agropecuaria, Hurlingham, Buenos Aires, Argentina.
[2]Consejo Nacional de Investigaciones Científicas y Técnicas, Ciudad Autónoma de Buenos Aires, Argentina
[3]Estación Experimental Agropecuaria INTA Balcarce, Balcarce, Buenos Aires, Argentina
[4]Bioinfomatics and Biostatistics Unit, Principe Felipe Research Center, Valencia, Spain
[5]Clinical Bioinformatics Area. Fundación Progreso y Salud (FPS). CDCA, Hospital Virgen del Rocio. 41013. Sevilla. Spain
[6]INB-ELIXIR-es, FPS, Hospital Virgen del Rocío, Sevilla, 42013, Spain.
†γThese authors contributed equally to this work
*Correspondence and requests should be addressed to MF (email: fass.monica@inta.gob.ar)

Supplementary Figures

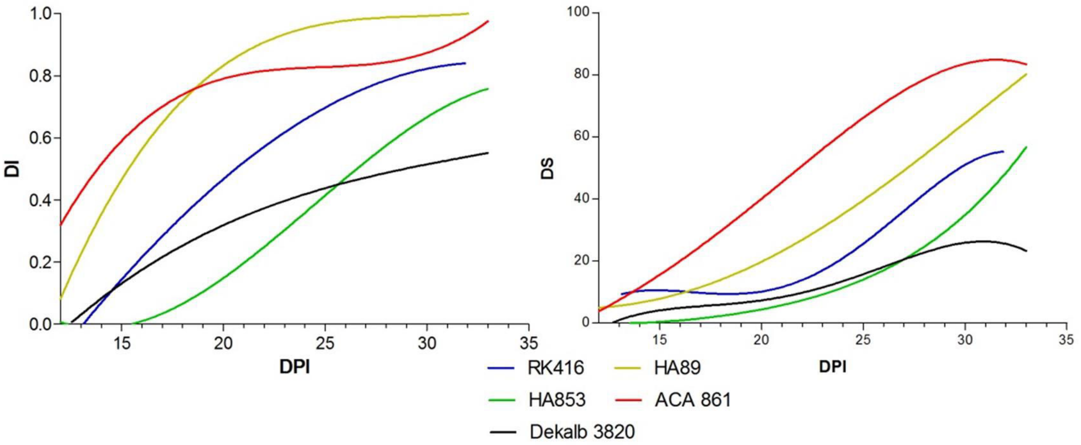

Supplementary Figure S1. Smoothed curves of progression of disease incidence (DI; left panel) and disease severity (DS; right panel) over time.

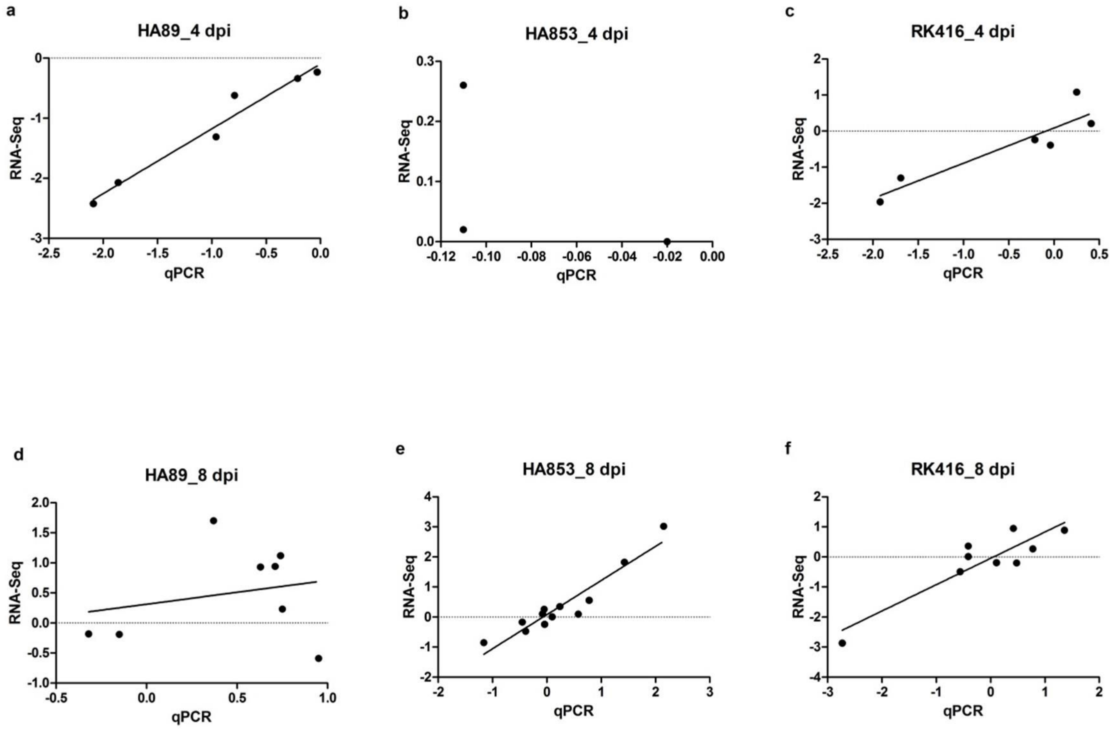

Supplementary Figure S2. Correlation of gene expression ratios between RNA-Seq and qPCR data. A, B, C: HA89, HA853 and RK416 at 4 dpi, respectively. D, E, F: HA89, HA853 and RK416 at 8 dpi, respectively.

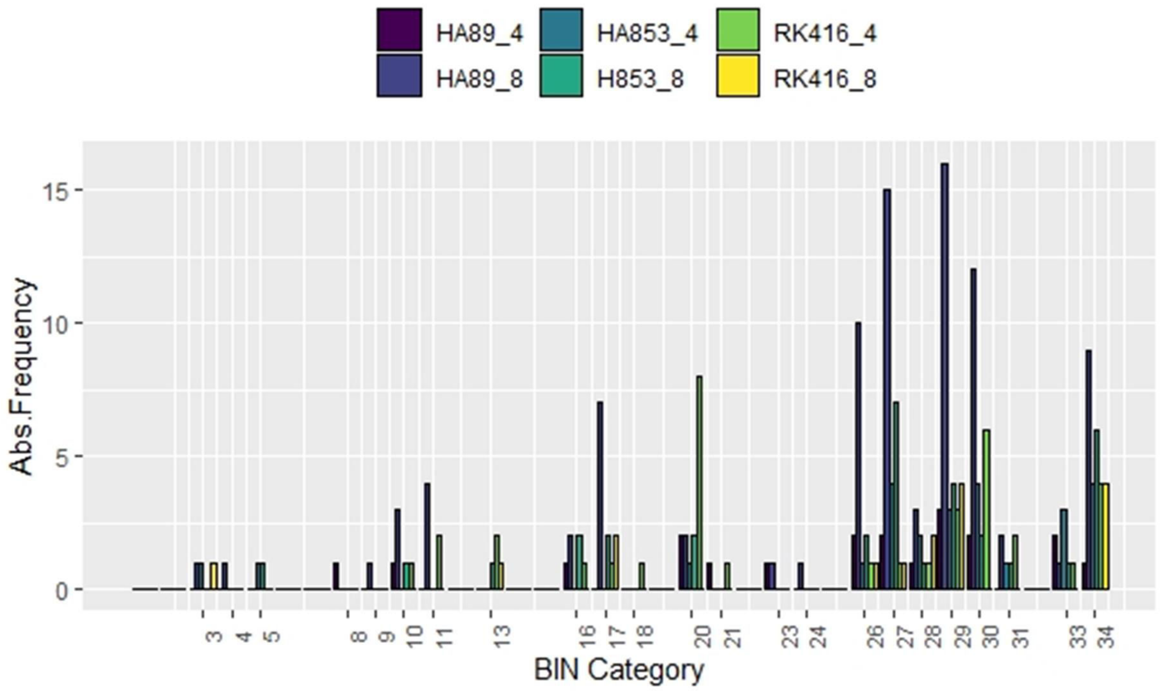

Supplementary Figure S3. Classification of DEGs of the different IL-time point combinations in MapMan categories. Numbers correspond to MapMan categories: 3: minor CHO metabolism; 4: glycolysis; 5: fermentation; 8: TCA / org transformation; 9: mitochondrial electron transport / ATP synthesis; 10: cell wall; 11: lipid metabolism; 13: amino acid metabolism; 16: secondary metabolism; 17: hormone metabolism; 18: Co-factor and vitamine metabolism; 20: stress; 21: redox; 23: nucleotide metabolism; 24: Biodegradation of Xenobiotics; 26: misc; 27: RNA; 28: DNA; 29: protein; 30: signalling; 31: cell; 33: development; 34: transport.
